# Controlled Structure of Electrochemically Deposited Pd Nanowires in Ion-Track Templates

**DOI:** 10.1186/s11671-015-1189-4

**Published:** 2015-12-12

**Authors:** Jinglai Duan, Shuangbao Lyu, Huijun Yao, Dan Mo, Yonghui Chen, Youmei Sun, K. Maaz, M. Maqbool, Jie Liu

**Affiliations:** Materials Research Center, Institute of Modern Physics, Chinese Academy of Sciences, Lanzhou, 730000 People’s Republic of China; University of Chinese Academy of Sciences, Beijing, 100049 People’s Republic of China; Nanomaterials Research Group, Physics Division, PINSTECH, Nilore, 45650 Islamabad Pakistan; Department of Physics and Astronomy, Ball State University, Muncie, IN 47306 USA

**Keywords:** Ion-track template, Palladium nanowires, Electrochemical deposition, Crystallinity, Crystallographic orientation

## Abstract

Understanding and controlling structural properties of the materials are crucial in materials research. In this paper, we report that crystallinity and crystallographic orientation of Pd nanowires can be tailored by varying the fabrication conditions during electrochemical deposition in polycarbonate ion-track templates. By changing the deposition temperature during the fabrication process, the nanowires with both single- and poly-crystallinities were obtained. The wires with preferred crystallographic orientations along [111], [100], and [110] directions were achieved via adjusting the applied voltage and temperature during electrochemical deposition.

## Background

Nanowire-based devices have been highly pursued for decades stimulated by their rapid expanding impact in nanotechnology. Metallic nanowires are not only interesting for fundamental research due to their unique structural and physicochemical properties compared to their bulk counterparts but also offer a fascinating potential for the future technological applications [1]. It is widely accepted that the performance of a given material is not only affected by its intrinsic properties but also influenced by the structural characteristics such as crystallinity and crystallographic orientation [2, 3]. For instance, the resistivity of a polycrystalline metal film can be enhanced dramatically due to electron scattering at the grain boundaries [4]. The hardness and yield stress of nanocrystalline materials typically increase with decreasing grain size, a phenomenon known as the Hall–Petch effect [5, 6]. However, in the case of very small grain sizes, the nanocrystalline copper becomes soft because of the plastic deformation originating from a large number of small “sliding” events of atomic planes at the grain boundaries, understood as anti-Hall–Petch effect [3, 7]. Similarly, the crystallographic orientation has great influence on the nanowires’ physicochemical properties. For example, the structural transition and melting of gold nanowires [8], the compressive pseudoelastic behavior in Cu nanowires [9], the thermal expansion of Cu nanowires [10], etc. Therefore, understanding and controlling structural properties of the nanowires have become a focus of the efforts to manipulate their electrical, mechanical, magnetic, and optical properties [3].

Among various noble metals, nanostructured Pd plays a significant role in many functional applications, such as hydrogen gas detection [11], catalyst in fuel cell [12], biosensors [13], and surface-enhanced Raman scattering [14]. In previous studies, Pd nanowires have been successfully fabricated by template-based strategies, like electrochemical deposition in anodic aluminum oxide (AAO) templates [15–22], chemical reaction in porous polycarbonate template [23], and soft template [24]. Some chemical methods, such as chemical reaction [13], lithographically patterned nanowire electrodeposition (LPNE) [25], galvanic displacement deposition [26], chemical vapor infiltration [27], and electroless deposition [28] have been employed to prepare Pd nanowires. Additionally, electron-beam lithography [29] and pattern-selective epitaxial growth [30] are also effective methods to fabricate Pd nanowires. In these studies, single crystalline [16, 23, 24, 30] and polycrystalline [21, 25] nanowires have been obtained by different experimental techniques and under different conditions. In these studies, single crystalline [16, 23, 24, 30] and polycrystalline [21, 25] nanowires have been obtained by different experimental techniques and under different conditions. Nanowires with preferred crystallographic orientations along the [111] and [110] directions have been reported by different research groups, however; these nanowires along [100] direction are hardly reported so far. To date, the challenge of controlling and tailoring the crystallinity and crystallographic orientation of Pd nanowires is still open.

In this work, we have prepared Pd nanowires by electrochemical deposition in homemade polycarbonate ion-track templates. Our results demonstrate that the Pd nanowires’ crystallinity and crystallographic orientation can be controlled by appropriately adjusting the fabrication conditions during the electrochemical deposition of the nanowires.

## Methods

In order to fabricate home-made ion-track templates, polycarbonate (PC) foils (Makrofol N, Bayer Leverkusen) with thickness of 30 μm were irradiated at the UNILAC linear accelerator of GSI (Darmstadt, Germany) with Pb ions (kinetic energy 11.4 MeV · u^−1^, fluence 5 × 10^8^ ions · cm^−2^) at normal incidence and at the HIRFL-SSC accelerator of IMP (Lanzhou, China) with Bi ions (kinetic energy 9.5 MeV · u^−1^, fluence 5 × 10^8^ ions · cm^−2^) at normal incidence. The damaged regions produced by the ions along their trajectories, called latent tracks, were selectively etched in 5 M NaOH at 50 °C leading to the formation of cylindrical nanopores in the foils. In this work, all PC foils were chemically etched for 3 min, corresponding to nanopores’ diameter of 75 nm. Prior to the etching, both sides of the foils were exposed to UV light for 2 h in order to enhance the selectivity of the etchant, and thus, to increase the track etching rate. This track sensitization is a necessary step to produce highly cylindrical nanopores. During the etching process, an ultrasonic field was employed to achieve a homogeneous etching.

The strategy to prepare Pd nanowires is based on ion-track template coupled with electrochemical deposition, which is described in detail elsewhere [31, 32]. First, a thin gold film was sputtered onto one side of the template that was further reinforced electrochemically by a Cu layer with a thickness of few microns. This back-layer (Au + Cu) served as cathode during the electrochemical deposition of the Pd nanowires. The electrolyte consisted of aqueous solution of 20 gl^−1^ K_2_PdCl_4_ and 20 gl^−1^ H_2_SO_4_. For fabrication of nanowires, direct current (DC) electrochemical deposition was employed with platinum rod used as the anode. The deposition process was monitored by recording current versus time curves. To make sure that the nanopores are completely filled, an overgrowth of nanowires was intentionally adopted, which resulted in the formation caps on the surface of the template.

After dissolving the polycarbonate templates in dichloromethane (CH_2_Cl_2_), the morphology, composition, and crystallinity of nanowires were investigated by means of scanning electron microscopy (SEM, JEOL JSM-6701F), transmission electron microscopy (TEM, JEOL JEM-3010), energy dispersive X-ray spectroscopy (EDS), and selected area electron diffraction (SAED). For TEM sample preparation, an ultrasonic field was used to detach the nanowires from the back-layer. The crystallographic orientations of the wire arrays were examined by X-ray diffraction (XRD, RIGAKU RINT 2400, Cu Kα, *λ* = 0.154056 nm). For XRD analysis, the wires were left embedded in the templates; however, both the caps and backing layer were removed from the nanowires.

## Results and Discussion

**Fig. 1:**
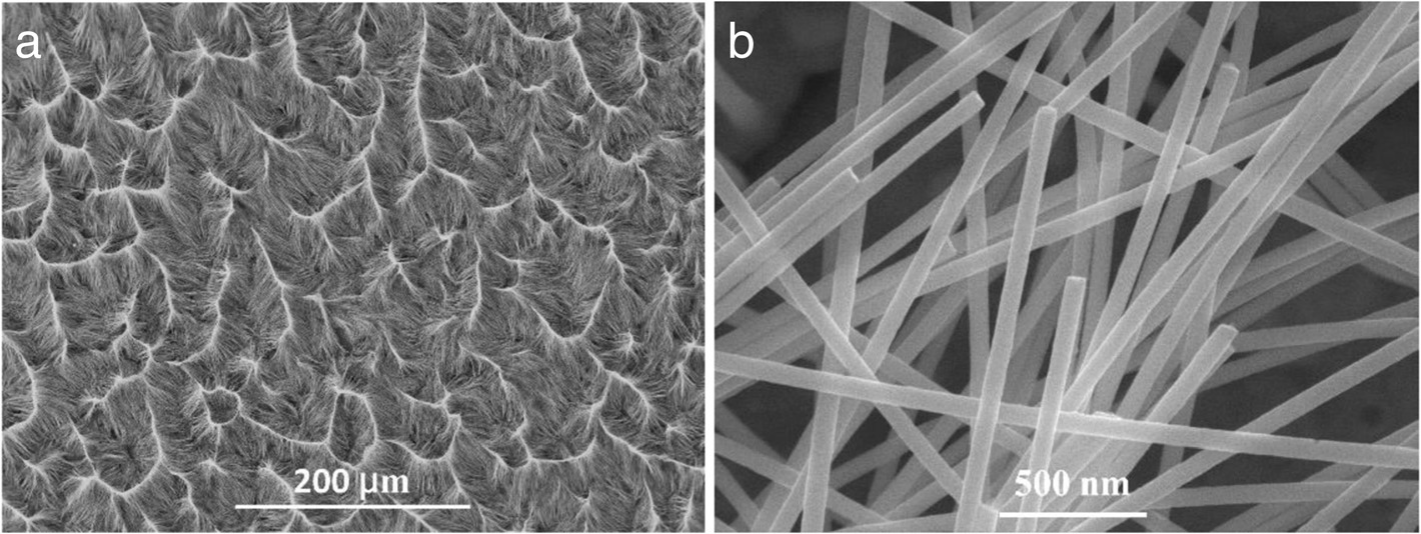
SEM images of the as-prepared Pd nanowires liberated from PC ion-track template at **a** low magnification and at **b** high magnification

The morphological characteristics of the prepared Pd nanowires were investigated by SEM, as demonstrated in Fig. 1. The SEM micrograph with low magnification, shown in Fig. 1a, shows that the wires are aggregated and homogeneously distributed on the backing layer after removing the template matrix by dichloromethane. The aggregation of nanowires originates from surface tension of dichloromethane droplets when drying the sample [33]. The high magnification SEM image shown in Fig. 1b reveals that the nanowires have perfect cylindrical shape with smooth and homogeneous contours along their length. The nanowires diameter is around 75 nm. Since the length of the wires is equal to template thickness (30 μm), the aspect ratio of the wires is expected to be as high as 400. For preparation of these nanowires, the applied voltage and deposition temperature were chosen as 0.8 V and 21 °C, respectively.

**Fig. 2:**
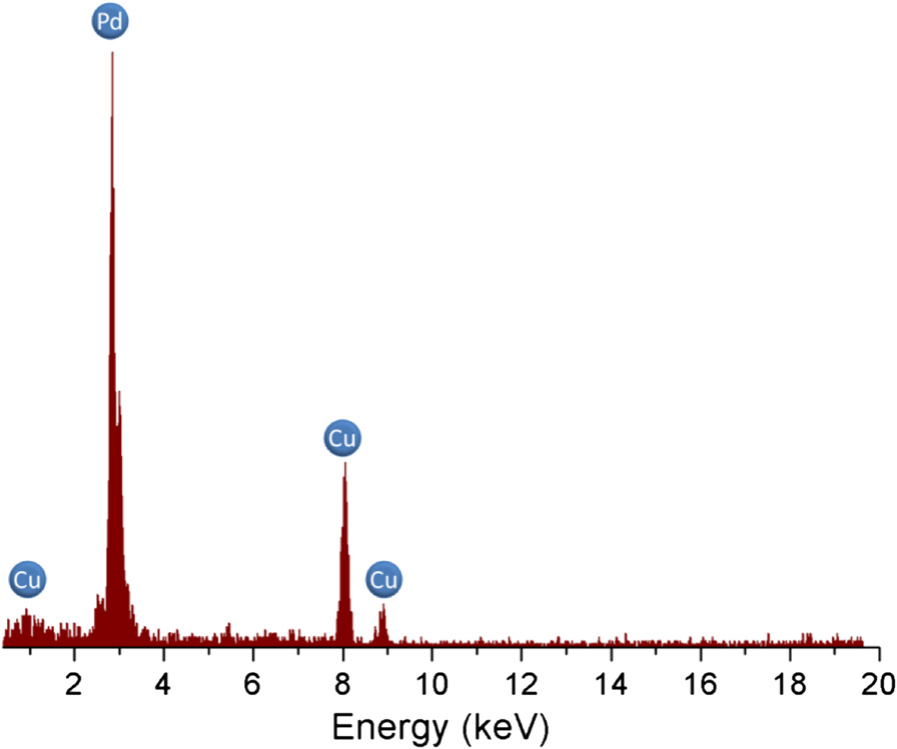
EDS spectrum of the as-prepared Pd nanowires

To examine the purity of the as-prepared Pd nanowires, the chemical composition of Pd nanowires was evaluated by TEM equipped with EDS. For TEM samples preparation, the wires were liberated from the host template using dichloromethane and subsequently transferred onto Cu TEM sample grid. The EDS spectrum of the nanowires is given in Fig. 2. It is obvious that, in addition to the Cu signals coming from the Cu grid, only Pd signals are seen in the spectrum, indicating that the nanowires are exclusively composed of Pd element.

**Fig. 3:**
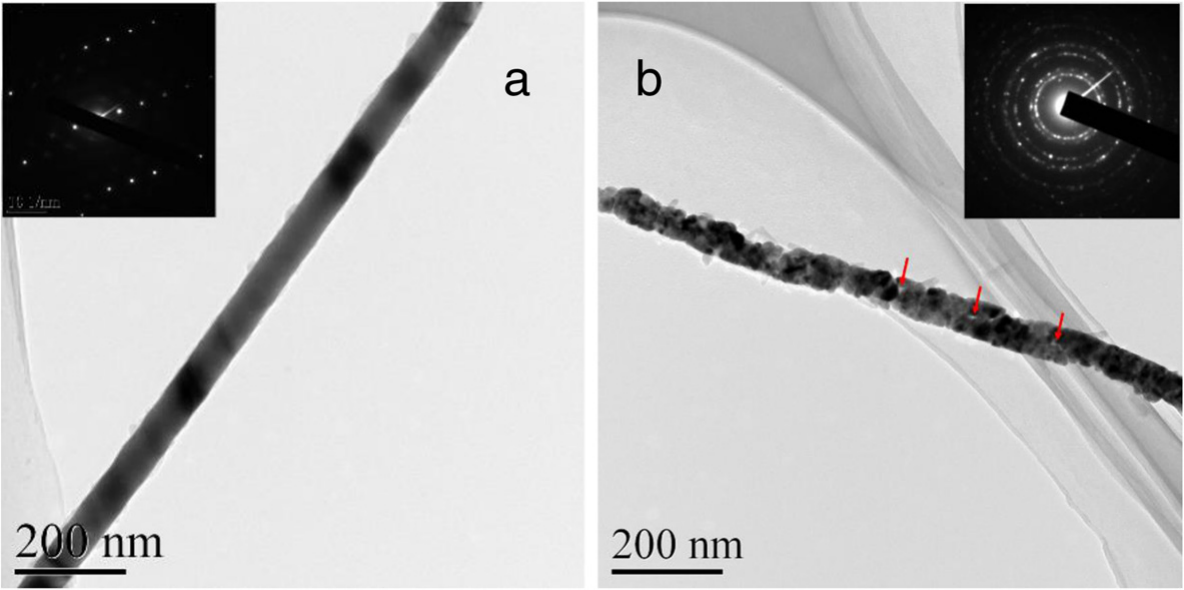
TEM images of the as-prepared Pd nanowires. **a** Single crystalline. **b** Polycrystalline. The *insets* are the corresponding SAED patterns

During the deposition, the crystallinity of Pd nanowires was controlled by the temperature which is one of the key parameters in electrochemical deposition [34]. Figure 3a shows the TEM image of Pd nanowires deposited at 21 °C, and Fig. 3b shows the TEM image of the wires deposited at 75 °C. The insets are the corresponding SAED patterns. For both samples, the same applied voltage of 1.4 V was used. For the wires deposited at 21 °C, as displayed in Fig. 3a, the surface of the wires is rather smooth and the diameter is homogeneous. These results are in good agreement with the SEM results. In addition, no grain boundaries are observed along the nanowire length, indicating the single crystalline nature of the nanowires. The dotted SAED pattern shown in the inset of Fig. 3a again supports this conclusion. However, for the wires deposited at 75 °C, the peripheral surface becomes rough and the nanowires seem to be comprised of small grains with some gaps within the grains are even observed, which are indicated by the arrows in Fig. 3b. The corresponding ring-like SAED pattern inserted in Fig. 3b convinces that the wires are polycrystalline. It is worthy to mention here that such polycrystalline nanowires with rough surfaces have more surface area and hence more surface energy as compared to single crystalline nanowires, and therefore, these nanowires would have better catalytic properties.

Two dimensional (2D) nucleation-growth mechanism and three dimensional (3D) nucleation-growth mechanism have been adopted to understand the growth mechanism of the electrochemically deposited nanowires and proposed to explain the formation mechanism of crystallinity of the electrochemically deposited nanowires [34, 35]. In both models, new grains will grow when the size of an initial grain nucleus exceeds the critical dimension *N*_*c*_ which is expressed as [34–36]:

1 $$ Nc=\frac{bs{\in}^2}{{\left(ze\eta \right)}^2} $$

for 2D growth, and

2 $$ Nc=\frac{8B{V}_m^2{\sigma}^3}{27{\left(ze\left|\eta \right|\right)}^3} $$

for 3D growth, where *b*(*B*), *s* (V_m_), ϵ (σ), *z*, and *e* are a constant (*b* = π for circular), the area (volume) occupied by one metallic atom on the surface of the nucleus, the edge (surface) energy, the effective electron number, and elementary charge (approximately 1.602176565(35) × 10^−19^ coulomb), respectively; *η* is the overpotential. From the formulae, it is obvious that lower *η* is favorable for the formation of single crystalline because *N*_*c*_ is larger. Otherwise, polycrystalline nanowires have more possibility to grow. Additionally, according to the formulae, only the overpotential *η* can be experimentally changed during the electrochemical deposition. Actually, the overpotential *η* is affected by the parameters like effective applied voltage and equilibrium potential. The effective applied voltage is the real applied voltage subtracting the voltage exhausted by electrolyte inside template channel because the electrolyte acted as a resistance. And the voltage exhausted by electrolyte inside template channel is greatly influenced by the temperature. Namely, higher temperature is beneficial for ions diffusion and, in sequence, making the resistance of electrolyte lower. In this case, the voltage exhausted by the electrolyte is less. Therefore, the overpotential *η* is higher and consequently the formation of polycrystalline nanowires is favorable, and the situation at lower temperature is vice versa.

**Fig. 4:**
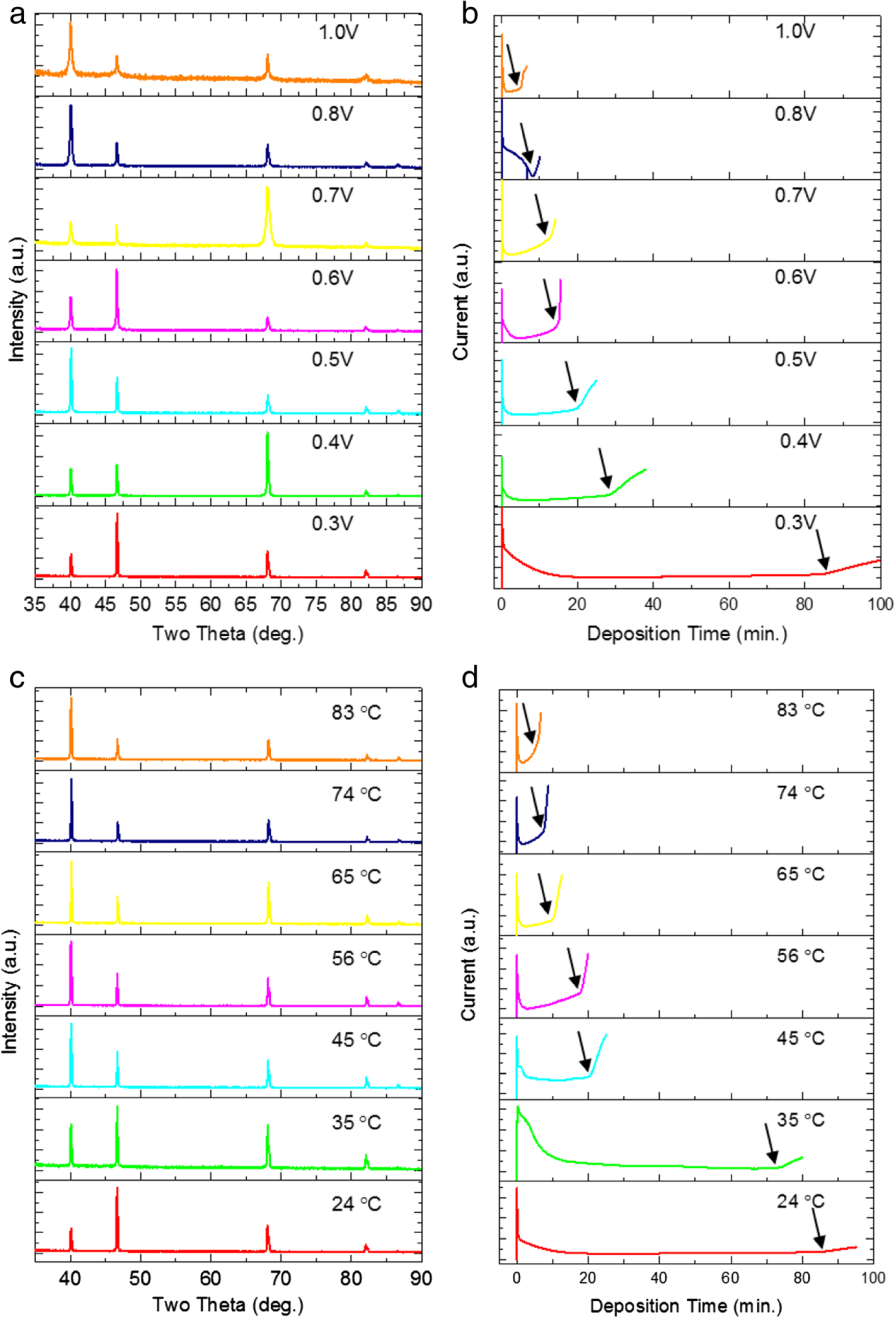
XRD patterns and corresponding current-time curves of the electrochemically deposited Pd nanowires. **a** XRD pattern of the wires deposited at applied voltages and constant temperature of 24 °C; **b** corresponding current-time curves of the wires shown in **a**; **c** XRD pattern of the wires deposited at different temperatures and a constant voltage 0.3 V; **d** corresponding current-time curves of the wires shown in **c**

Applied voltage and deposition temperature are the parameters adopted to tailor the nanowires crystallographic orientations in this work. Figure 4a, b display the XRD patterns of Pd nanowire arrays fabricated at different temperatures and applied voltages, respectively. For XRD measurements, the nanowires were kept embedded in templates after the removal of back-layer as described in the experimental section. As seen in Fig. 4a, the relative intensities of the diffraction peaks of (111), (200), and (220) planes change dramatically with increasing applied voltage, whereas the temperature was kept at 24 °C throughout. In cases of 0.5 V, 0.8 V, and 1.0 V, the (111) peak is the most intensive among the peaks. For the wires deposited at 0.3 V and 0.6 V, the (200) peak intensities become the strongest. The (220) peaks become the most intensive when the applied voltage was changed to 0.4 V and 0.7 V. The electrochemical deposition processes were monitored by the current-time (*I-t*) curves [37]. Figure 4b shows the recorded *I-t* curves of the wires shown in Fig. 4a. It is seen that, with increasing applied voltage, the time of current rise is gradually short (indicated by arrows), revealing that the nanowire growth rate is accelerated at larger voltages. In Fig. 4c, the relative intensities of the diffraction peaks of (111), (200), and (220) planes change remarkably with increasing temperature, whereas the applied voltages were kept constant at 0.3 V. For the nanowires deposited at lower temperatures (24 and 35 °C), the (200) peak is the strongest one, indicating the growth orientation along the [100] direction. However, at higher temperatures (45–83 °C), the (111) peak becomes the most intensive, revealing the growth orientation is along [111] direction. Figure 4d shows the recorded *I-t* curves of the wires shown in Fig. 4c. At elevated temperatures, the nanowire growth rate is significantly enhanced, as indicated by arrows.

The electrochemical deposition of metallic nanowires is a complex process, which is influenced by the factors such as charge transfer, ions diffusion, and H ions or micelles absorption. However, from the energy point of view, the crystal plane with low surface energy has higher possibilities to grow and fulfill the principle of minimization of free energy. It has been reported that H ions absorption on the crystal planes could decrease the surface energies of the crystal planes and such absorption on different crystal planes can be affected by overpotential [3, 38]. In this work, our electrolyte consisted of H_2_SO_4_ which provided H ions during the wires growth. Additionally, a high overpotential may give rise to the surface adsorption of H ions or micelles in the electrolyte and thus considerably enhances the electrochemical reaction driving force, which increases the deposition rate of metallic ions during the deposition [37], as shown in Fig. 4b. At different overpotentials, the surface energies of the (111), (200), and (220) planes changed via H ions absorption. The surface energies of the (111), (200), and (220) planes reached their minimum values when appropriate voltages were applied. Therefore, the crystallographic orientations along [111], [100], and [110] directions are obtained by changing the applied voltage during deposition process. The application of high temperature promotes the surface diffusion of the atoms and reduces the minimum applied voltage required to grow Pd wires and consequently increases the growth rate of the nanowires (Fig. 4d). Therefore, temperature is another factor that influences the free energy of Pd nanowires during the electrochemical deposition process via changing H ions adsorption, resultantly influences the crystallographic orientation of Pd nanowires.

## Conclusions

Pd nanowires have been successfully fabricated in home-made polycarbonate ion-track templates by electrochemical deposition. The Pd nanowires with single crystalline and polycrystalline structures have been obtained via changing electrochemical deposition temperature. The critical grain size model is adopted to explain the effect of temperature on Pd nanowires’ crystallinity. The crystallographic orientations of the Pd nanowires along [111], [100], and [110] directions have been achieved and can be controlled by the applied voltage and temperature during the electrochemical deposition. A possible mechanism based on H ions absorption has been proposed to understand the control over the nanowires crystallographic orientations.
